# Effects of Marathon Running on Aerobic Fitness and Performance in Recreational Runners One Week after a Race

**DOI:** 10.1155/2017/9402386

**Published:** 2017-08-24

**Authors:** Fuminori Takayama, Atsushi Aoyagi, Wataru Shimazu, Yoshiharu Nabekura

**Affiliations:** ^1^Graduate School of Comprehensive Human Sciences, University of Tsukuba, 1-1-1 Tennodai, Tsukuba, Ibaraki 305-8574, Japan; ^2^Japan Society for the Promotion of Sciences, 5-3-1 Kojimachi, Chiyoda-ku, Tokyo 102-0083, Japan; ^3^Faculty of Health and Sport Science, University of Tsukuba, 1-1-1 Tennodai, Tsukuba, Ibaraki 305-8574, Japan

## Abstract

It is not clear whether or not recreational runners can recover aerobic fitness and performance within one week after marathon running. This study aimed to investigate the effects of running a marathon race on aerobic fitness and performance one week later. Eleven recreational runners (six men, five women) completed the race in 3 h 36 min 20 s ± 41 min 34 s (mean ± standard deviation). Before and 7 days after the race, they performed a treadmill running test. Perceived muscle soreness was assessed before the race and for the following 7 days. The magnitude of changes in the treadmill running test was considered* possibly trivial* for maximal oxygen uptake ($\dot{V}$O_2_max) (mean difference −1.2 ml/kg/min; ±90% confidence limits 2 ml/kg/min),* unclear* for %$\dot{V}$O_2_max at anaerobic threshold (AT) (−0.5; ±4.1%) and RE (0.2; ±3.5 ml/kg/km), and* likely trivial* for both velocity at AT and peak (−0.2; ±0.49 km/h and −0.3; ±0.28 km/h). Perceived muscle soreness increased until 3 days after the race, but there were no clear differences between the values before the race and 4–7 days after it. These results show that physiological capacity associated with marathon running performance is recovered within 7 days after a marathon run.

## 1. Introduction

Marathon running (running for 42 km) is a popular form of vigorous physical activity [1]. Whatever the runner's level of ability, marathon running places an increased workload on their physiological function over the course of several hours. A previous study showed that during a marathon race the fractional use of maximal heart rate ranged from about 80% to 90% [2], suggesting that cardiorespiratory strain is high.

The number of recreational marathon runners has increased over the last decade [3]. Some runners participate in consecutive weekend races [4]. The high frequency of races that do not allow for sufficient recovery time may lead to causing overtraining syndrome. Overtraining has been defined as excessive stress without adequate rest or recovery period, which results in performance decrements with or without related change physiological sign [5].

Among the physiological characteristics, maximal oxygen uptake ($\dot{V}$O_2_max), fractional utilization of $\dot{V}$O_2_max (%$\dot{V}$O_2_max, determined from anaerobic threshold: AT), and running economy (RE) have been used as the classic model of predicting distance running performance [6]. In addition to these measures of aerobic fitness, both velocity at AT and peak during a treadmill running test are used as indirect measurement of long distance running performance [7, 8]. For example, Noakes et al. demonstrated a strong correlation between peak velocity and marathon time (*r* = −0.89, *p* < 0.01) in marathon runners (range of race time: 2 h 08 min–3 h 26 min) [7].

Recovery is typically quantified as ability to meet or exceed preexercise performance in a particular activity [9]. There is a relative lack of information on recovery of aerobic fitness and performance. Among the limited studies on this topic, Kyröläinen et al. investigated $\dot{V}$O_2_ during submaximal running before and for the 6 days after marathon running and reported that it took 4–6 days to recover to the condition before marathon running [10]. This result implied that submaximal aerobic fitness could be recovered within one week after a race. For maximal aerobic fitness, Noble et al. showed no significant difference in $\dot{V}$O_2_max between the value before the race and 2 weeks after it [11]. On the other hand, most recent evidence has indicated that peak oxygen uptake was significantly lower 3 to 4 days after a marathon than it was before the race, suggesting that marathon running reduces maximal aerobic fitness for the first few days after a race [12]. However, we are unaware of any studies that have tried simultaneously to elucidate the effects of marathon running on $\dot{V}$O_2_max, %$\dot{V}$O_2_max, and RE after a race. Thus, it remains unclear whether or not a runner can completely recover aerobic fitness and performance within one week after marathon running. Marathon races are generally held every weekend, and it is therefore extremely important for recreational runners to understand recovery status a week after marathon running.

The purpose of the present study was to investigate the effects of marathon running on aerobic fitness and performance one week after a race. We hypothesized that marathon running would result in no differences in physiological characteristics a week after a race.

## 2. Materials and Methods

### 2.1. Participants

Eleven recreational runners (6 males, 5 females) participated in the study. The inclusion criteria were as follows: (1) the subjects regularly trained at least three days per week for a marathon race; (2) the subjects were healthy and reported no cardiorespiratory or musculoskeletal disorders; (3) the subjects were of an adult age; and (4) the subjects were nonsmokers. Before participation, the participants were provided with information sheets about the study process, and they provided written informed consent and completed a questionnaire regarding their training status. The physical characteristics and training status of the participants are shown in Table 1. This study was approved by the Ethical Committee of the University of Tsukuba (Ref number Tai 27–76).

### 2.2. Experimental Design

All measurements were made before and after the Tsukuba Marathon held in Ibaraki, Japan. To increase sample size, the present study was conducted over a 2-year period. Eight subjects participated in the race 2015 and three subjects participated in the race 2016. The race was officially recognized by the Japan Association of Athletics Federations and a flat-road race course. Both races were cloudy and had similar conditions: the temperature was 12.4°C and 10.1°C, the relative humidity 68% and 98%, and wind speed 2.5 m/s and 2.0 m/s, respectively, at the start of the race.

To investigate the effects of marathon running on physiological characteristics one week after the race, a treadmill running test was performed 1-2 weeks before (PRE) and 7 days after (POST) the marathon by the same investigators. Individual tests were performed at the same time of day (±1 h) to avoid any diurnal variation effects. In addition, perceived muscle soreness was recorded before and for the 7 days after the race.

### 2.3. Treadmill Running Test

The subjects were familiarized with treadmill running before the PRE measurement. The test was performed on a motorized treadmill (ORK-7000, Ohtake Root Kogyo, Japan) set at 1% grade, which accurately reflects the energetic cost of outdoor running [13]. The subjects consumed a light meal at least 3 h before the test, and during the 3 h preceding the test only ad libitum water intake was allowed. Body weight was measured before the test (TBF-102, Tanita, Japan).

The subjects completed a two-part test that consisted of RE test and a maximal incremental test. Expired gas analysis was continuously performed on a breath-by-breath basis using the computerized standard open circuit technique, and the data were then converted into 20-s time binned mean values (AE-310s, Minato Medical Science, Japan). Before each test, the oxygen and carbon dioxide analysers were calibrated using known gas concentrations and flow calibration was performed using a 2-L syringe. HR was collected via telemetry (Polar, Finland).

The subjects underwent a 5-min RE test at submaximal intensity. The intensity was set for each subject based on their questionnaire answer about their target time for the race. As RE can be assessed by $\dot{V}$O_2_ in steady state submaximal running [14], we set the treadmill velocity at 85% of the velocity corresponding with the target time of the race. The gas analysis data during the last 1-min were used in the analysis. RE was expressed as O_2_ cost (ml/kg/km).

Following a 5-min recovery period after the RE test, the maximal incremental test was performed to determine $\dot{V}$O_2_max, AT, and peak velocity. The initial velocity was set at 8.4 km/h and it was increased by 0.6 km/h every 60 s until volitional exhaustion, defined as the point at which the subject could no longer run at the required velocity. The subjects were always verbally encouraged by the same investigators. To avoid external feedback, the subjects were unable to see the displays of the expired gas analysis and heart rate. AT was defined as the point when respiratory exchange ratio (RER) stabilized above 1.0 not returning to levels below [15]. The AT was expressed as velocity and %$\dot{V}$O_2_max (%$\dot{V}$O_2_max at AT). $\dot{V}$O_2_max was defined by the attainment of at least three of the following four criteria: (1) a leveling off in $\dot{V}$O_2_ despite an increase in a treadmill velocity, (2) RER ≥ 1.1, (3) HR ≥ 90% of the subject's age-predicted HRmax (i.e., 220 – age), and (4) peak RPE ≥ 19. Peak velocity was determined by the last stage of the test. When the subject was unable to complete a full 60 s at the required velocity, the peak velocity was determined as a fraction of the final velocity added to the velocity in the immediately preceding 60 s.

### 2.4. Perceived Muscle Soreness

Perceived muscle soreness was assessed using a verbal rating scale of 0 (no pain) to 10 (extreme pain) [16]. Before and 7 days after running the marathon, perceived muscle soreness was determined before undertaking the treadmill running test, whereas it was assessed every evening for the 6 days after the marathon. The subjects were instructed to record the soreness levels of their knee extensors, knee flexors, plantar flexors, hips, upper back, lower back, shoulders, elbow flexors, and elbow extensors on a questionnaire sheet while stretching specific muscles.

### 2.5. Statistical Analysis

The statistical analyses were performed with SPSS Statistics 22 (IBM Japan, Japan). Data are expressed as mean and SD. Because recovery is typically quantified as the ability to meet or exceed preexercise performance in a particular activity [9], we conducted paired *t*-tests to estimate the post-pre difference in outcomes. Perceived muscle soreness was analyzed by separate one-way repeated-measures analyses of variance (ANOVAs) and Dunnett post hoc tests. Assumptions of sphericity were assessed using Mauchly's test, with any violations adjusted via the Greenhouse–Geisser correction. The data were analyzed for practical significance using Cohens' *d* effect sizes (ES) and magnitude-based inferences. The ES were classified as trivial (<0.2), small (0.2–0.6), moderate (0.6–1.2), large (1.2–2.0), and very large (>2.0) [17]. Ninety percent confidence intervals for between PRE and POST differences in changes were estimated, and magnitude-based inferences were made with reference to the smallest worthwhile change (SWC), which was calculated as being 0.2 between-subject standard deviation of the PRE value. Quantitative chances of higher or lower differences than SWC were evaluated qualitatively as follows: <0.5%,* most unlikely* or* almost certainly not*; 0.5% to 5%,* very unlikely*; 5% to 25%,* unlikely* or* probably not*; 25% to 75%,* possibly*; 75% to 95%,* likely* or* probably*; 95% to 99.5%,* very likely*; >99.5%,* most likely* or* almost certainly*. If the chance of an increase and decrease effect were both >5%, the true difference was considered to be* unclear* [17].

Relationships between both velocity at AT and peak and the average velocity during the marathon were determined using Pearson's product–moment correlation coefficient tests. Simultaneous multiple regression analysis was used to predict performance from a classic model that included $\dot{V}$O_2_max, %$\dot{V}$O_2_max at AT, and RE.

## 3. Results

The subjects completed the race in 3 h 36 min 20 s ± 41 min 34 s (range: 2 h 31 min 22 s to 4 h 54 min 48 s), which is similar to the combined target time for the race (3 h 37 min 38 s ± 54 min 02 s, *p* > 0.05). The subjects' average velocity during the marathon was 12.1 ± 2.4 km/h.

### 3.1. Treadmill Running Test

Table 2 shows the changes in aerobic fitness and indirect performance from PRE to POST. The mean velocity in the RE test was 10.5 ± 2.6 km/h. In the RE test, the RER of all subjects were less than 1.00, both PRE and POST. For all three variables, no significant differences or trivial effect sizes were observed between values measured PRE and POST. The magnitude of changes was considered* possibly trivial* for $\dot{V}$O_2_max,* unclear* for %$\dot{V}$O_2_max and RE, and* likely trivial* for both velocity at AT and peak. Significant correlations were found between the subject's average velocity during the marathon and PRE values of AT (km/h) (*r* = 0.899; *p* < 0.001) and peak velocity (*r* = 0.916; *p* < 0.001). Similarly, the average velocity during the marathon was significantly correlated with the POST values of AT (*r* = 0.897; *p* < 0.001) and peak velocity (*r* = 0.907; *p* < 0.001). Simultaneous multiple regression analysis was used to determine whether the classic model could predict average velocity during the marathon. A significant prediction equation was found (*F* = 15.363, *p* = 0.002) that accounted for 87% of the variance in average velocity during the marathon (*R*^2^ = 0.87) at PRE measurement. In the same way, a significant prediction was found (*F* = 12.450, *p* = 0.003) that accounted for 84% of the variance in average velocity during the marathon (*R*^2^ = 0.84) at POST measurement.

### 3.2. Perceived Muscle Soreness

Table 3 shows the changes in perceived muscle soreness. Separate one-way ANOVAs demonstrated significant main effects of day for the perceived muscle soreness of all muscles. The increases in knee extensors were* most likely* at 1 and 2 days after the marathon and* very likely *at 3 days. The increases in knee flexors and hips were* most likely* at 1 and 2 days after the marathon. The increases in plantar flexors, upper back, and lower back were* most likely* at 1 day after the marathon and* very likely* at 2 days after. The increases in shoulders were* most likely* at 1 day after the marathon and* likely* at 2 days after. The increases in elbow flexors were* likely* at 1 day after the marathon. However, there were no clear differences in any variables between before and 4–7 days after the marathon.

## 4. Discussion

The main determinants of marathon running performance are aerobic fitness [6]. Previous study has shown that classic model ($\dot{V}$O_2_max, %$\dot{V}$O_2_max, and RE) explains >70% of variance in distance running performance [18]. In the present study, classic model explained more than 80% of variance in average velocity during marathon. Moreover, the significant relationships between performance variables and marathon performance were similar to those reported in previous study [7]. Thus, the treadmill running test used in the present study was an effective method for predicting marathon running performance.

To the best of our knowledge, the present study is the first to systematically investigate the effects of marathon running on aerobic fitness one week after a marathon race. We found no significant differences between the values of $\dot{V}$O_2_max, %$\dot{V}$O_2_max at AT, and RE measured before and 7 days after completing a marathon. Moreover, the results of magnitude-based inferences showed that marathon running does not have an adverse effect on aerobic fitness and performance seven days after the race. As mentioned earlier, recovery is typically quantified as ability to meet or exceed preexercise performance in a particular activity [9]. Based on the results of the present study, the examined physiological characteristics related to marathon running performance can recover within one week after running a marathon in a sample of recreational runners.

It is well known that marathon running induces delayed-onset muscle soreness (DOMS) [19, 20], which is one of the main symptoms of exercise induced muscle damage (EIMD). Not only is DOMS an indication of subclinical injury [21] but it also has a negative impact on aerobic fitness [22, 23] and performance [24] during running. For example, Braun and Dutto investigated changes in $\dot{V}$O_2_ during steady state submaximal running and muscle soreness before and 2 days after downhill running and showed that $\dot{V}$O_2_ and muscle soreness were significantly elevated 2 days after the exercise [23]. Based on this result, they noted changes in RE due to downhill running-induced muscle damage. Another study has also reported that peak oxygen uptake and velocity at ventilatory threshold were reduced and perceived muscle soreness increased 7 days after single-leg split squats with 40% of body weight [22]. In comparison with the previous study [22], the magnitude of changes in physiological characteristics and perceived muscle soreness was milder in the present study. Although we did not measure other muscle damage markers such as creatine kinase activity and maximal voluntary isometric contraction torque, it has clearly been demonstrated in many studies that muscle damage markers can recover within one week after marathon running [10, 19, 20, 25]. Taken together, we conclude that no differences in aerobic fitness and distance running performance result from the recovery from EIMD within one week after running a marathon.

There were some limitations to the present study. First, the study was descriptive and neither the training nor the food intake after marathon running was controlled. Previous studies suggested that some recovery methods, such as low intensity running [26] and the ingestion of branched-chain amino acids [27] and tart cherry juice [28], have an impact on the recovery of muscle damage after marathon running. Therefore, the varying activities performed by each individual after the race may have introduced some bias into the study results. Second, our sample was relatively small and of mixed-sex. However, the treadmill running test is highly reliable for detecting small changes [29]. Moreover, both men and women have been shown to respond similarly to EIMD [30]. Third, it should be noted that the present study included only recreational runners and had no corresponding control group. Finally, we performed the treadmill running test only once after the subject had run a marathon. Further studies of the time course of recovery of physiological characteristics are needed to fully establish the recovery status after marathon running in runners with various levels of running ability such as elite runners.

## 5. Conclusion

In conclusion, this study estimated the effects of marathon running on aerobic fitness and performance one week after a marathon race. The results suggest that marathon running does not adversely affect a person's aerobic fitness and performance 7 days after the race. Based on the results of this study, we suggest that maximal aerobic performance capability and measure of threshold and economy are restored within one week after running a marathon.

## Figures and Tables

**Table 1:** Physical characteristics and training status of the subjects.

	Mean ± SD
Age (years)	24 ± 4
Height (cm)	168 ± 9
Weight (kg)	62.0 ± 9.8
Running experience (years)	6 ± 4
Training distance during the 1 month before the race (km)	215 ± 145

**Table 2:** Changes in aerobic fitness before and seven days after a marathon race.

	PRE	POST	*p* value	Mean difference; 90% CL	Effect size	Qualitative inference
O_2_ cost (ml/kg/km)	221.0 ± 14.9	221.2 ± 11.2	0.919	0.2; ±3.5	0.02	Unclear
%$\dot{V}$O_2_max at AT (%)	85.8 ± 4.7	85.3 ± 4.6	0.830	−0.5; ±4.1	−0.10	Unclear
$\dot{V}$O_2_max (ml/kg/min)	59.3 ± 9.5	58.1 ± 9.2	0.294	−1.2; ±2	−0.12	Possibly trivial
AT (km/h)	14.6 ± 2.6	14.4 ± 2.3	0.476	−0.2; ±0.49	−0.09	Likely trivial
Peak velocity (km/h)	17.2 ± 2.5	16.9 ± 2.5	0.082	−0.3; ±0.28	−0.09	Likely trivial

**Table 3:** Perceived muscle soreness before and for seven days after a marathon race.

	Before	1 day	2 days	3 days	4 days	5 days	6 days	7 days
Knee extensors	1.0 ± 1.3	6.1 ± 2.2^*∗∗∗∗*^	4.8 ± 2.2^*∗∗∗∗*^	2.8 ± 1.9^*∗∗∗*^	1.9 ± 1.2	1.1 ± 0.9	1.2 ± 1.1	0.7 ± 1.1
Knee flexors	1.4 ± 1.2	5.2 ± 2.4^*∗∗∗∗*^	4.0 ± 2.2^*∗∗∗∗*^	2.5 ± 1.8	1.5 ± 1.1	1.2 ± 1.1	1.3 ± 1.0	0.7 ± 1.1
Plantar flexors	1.7 ± 2.2	4.9 ± 2.0^*∗∗∗∗*^	3.5 ± 2.1^*∗∗∗*^	1.9 ± 1.5	1.4 ± 1.3	1.3 ± 1.1	1.2 ± 1.0	0.6 ± 1.0
Hips	0.9 ± 1.4	4.0 ± 2.9^*∗∗∗∗*^	2.9 ± 2.3^*∗∗∗∗*^	1.6 ± 1.4	1.3 ± 1.3	1.2 ± 1.5	1.1 ± 1.1	0.9 ± 1.3
Upper back	0.5 ± 0.8	1.8 ± 1.9^*∗∗∗∗*^	1.5 ± 1.4^*∗∗∗*^	0.5 ± 0.8	0.4 ± 0.7	0.3 ± 0.6	0.5 ± 1.0	0.3 ± 0.6
Lower back	0.7 ± 1.0	2.6 ± 2.6^*∗∗∗∗*^	2.0 ± 2.2^*∗∗∗*^	1.2 ± 2.0	0.8 ± 1.8	0.7 ± 1.7	0.6 ± 1.2	0.5 ± 0.8
Shoulders	0.9 ± 1.6	2.8 ± 2.6^*∗∗∗∗*^	1.9 ± 2.0^*∗∗*^	0.8 ± 1.0	0.5 ± 0.8	0.5 ± 0.8	0.6 ± 1.1	0.5 ± 0.8
Elbow flexors	0.7 ± 1.6	1.8 ± 1.8^*∗∗*^	0.8 ± 1.3	0.5 ± 0.8	0.3 ± 0.6	0.1 ± 0.3	0.2 ± 0.4	0.1 ± 0.3
Elbow extensors	0.6 ± 1.6	1.2 ± 1.7	0.5 ± 1.0	0.2 ± 0.6	0.2 ± 0.6	0.1 ± 0.3	0.1 ± 0.3	0.2 ± 0.6

Qualified likelihood was shown as increased number of symbols: ^*∗*^possible, ^*∗∗*^likely, ^*∗∗∗*^very likely, and ^*∗∗∗∗*^most likely.
